# Supplementary material for: Hyperbaric oxygen in children with cerebral palsy: A systematic review of effectiveness and safety
Source: PLoS One. 2022 Oct 14;17(10):e0276126. doi: 10.1371/journal.pone.0276126 (PMC9565562; doi:10.1371/journal.pone.0276126)
Supplement: Attachment — Submitted filename: Comment for Hyperbaric Oxygen_review.doc [file pone.0276126.s005.doc]

**Comments**

**General comments**

As there are a number of omissions in the design of this study, the value of this topic under review is questionable.

**Abstract/ Introduction-content**

No read

**Methods: Identification and selection of studies**

Information is messy. Inclusion criteria should be following as Design(missing), Participants, Intervention (missing), Outcome measures (missing)

**Methods: Assessment of characteristics of studies**

There should be more points in this part. It would be better to have this headings and follow some subheading as below:

*Quality*: information included , but a bit messy.

*Participants:* missing

*Intervention:* messing

*Outcome measures:* missing

**Results/ Discussion/ Conclusion: context.**

As there are so many questions in the method, results, discussion and conclusion are questionable.

**Readability and style.**

This paper is not easy to read, because the fluency and structure of written English, suggesting for proofreading.
