# Supplementary material for: Hyperbaric oxygen in children with cerebral palsy: A systematic review of effectiveness and safety
Source: PLoS One. 2022 Oct 14;17(10):e0276126. doi: 10.1371/journal.pone.0276126 (PMC9565562; doi:10.1371/journal.pone.0276126)
Supplement: Attachment — Submitted filename: Responses to reviewers.doc [file pone.0276126.s006.doc]

**Comments**

**General comments**

As there are a number of omissions in the design of this study, the value of this topic under review is questionable.

We have strictly followed the PRISMA guidelines for systematic reviews and meta-analyses and the bodies of evidence were then appraised using the GRADE evidence rating system, which ensures that the methods and results of the present review meet the stat-of-the-art expectations.

**Abstract/ Introduction-content**

No read.

We assume that there has been no detectable issue in this section.

**Methods: Identification and selection of studies**

Information is messy. Inclusion criteria should be following as Design(missing), Participants, Intervention (missing), Outcome measures (missing)

We followed the reviewer’s demand and altered this paragraph of the Methods section, which now reads:

“The following inclusion criteria were used for study selection:

1) Design: observational studies, before/after studies (descriptive) and controlled trials
2) Patients: children (<18 years) with CP included in the study sample;
3) Intervention: administration of HBOT or HBA;
4) Outcomes: inclusion of an effectiveness- and/or safety-related outcome for HBOT or HBA;
5) Language: articles written in English.”

The title of the paragraph has been changed into the following: *Article selection: Identification and selection of studies.*

**Methods: Assessment of characteristics of studies**

There should be more points in this part. It would be better to have this headings and follow some subheading as below:

*Quality*: information included , but a bit messy.

*Participants:* missing

*Intervention:* messing

*Outcome measures:* missing

We have taken this comment of the reviewer into account and added the suggested organization in the Methods section. We added the following subheadings:

*“Participants:* information about participants (age, type of cerebral palsy, and functional level) were detailed.

*Intervention:* data regarding the hyperbaric intervention (content: pure oxygen or air, pressure, number and duration of sessions, total volume of the intervention) and control intervention were carefully searched through and reported.

*Outcome measures:* all types of outcome measures identified in the retained articles were analysed and could subsequently be sorted as: motor function, spasticity, sleep disorders, cognitive functions, independence, for effectiveness-related outcome, and safety.”

Besides, the title of this paragraph of the Methods section has been changed and now reads: “***Quality assessment and study appraisal : Assessment of the characteristics of included studies*** in order to provide more information about the methods used.”

**Results/ Discussion/ Conclusion: context.**

As there are so many questions in the method, results, discussion and conclusion are questionable.

We are very confident that all informations about the methods that were missing in the reviewer’s opinion have been detailed in the Results section and the associated Tables

**Readability and style.**

This paper is not easy to read, because the fluency and structure of written English, suggesting for proofreading.

We asked for a careful assessment of the English in this manuscript by a native English speaker.
