# Supplementary material for: Hyperbaric oxygen in children with cerebral palsy: A systematic review of effectiveness and safety
Source: PLoS One. 2022 Oct 14;17(10):e0276126. doi: 10.1371/journal.pone.0276126 (PMC9565562; doi:10.1371/journal.pone.0276126)
Supplement: Attachment — Submitted filename: 2_Comment for Hyperbaric Oxygen_review.doc [file pone.0276126.s007.doc]

**Comments**

**General comments**

As there are a number of omissions in the design of this study, the value of this topic under review is questionable. Authors still didn’t change methods as suggested. It would be difficult to read futher.

**Abstract/ Introduction-content**

No read

**Methods: Identification and selection of studies**

Information is messy. Inclusion criteria should be following as Design(missing), Participants, Intervention (missing), Outcome measures (missing)

**Methods: Assessment of characteristics of studies**

There should be more points in this part. It would be better to have this headings and follow some subheading as below:

*Quality*: information included , but a bit messy.

*Participants:* missing

*Intervention:* messing

*Outcome measures:* missing

**Results/ Discussion/ Conclusion: context.**

As there are so many questions in the method, results, discussion and conclusion are questionable.

**Readability and style.**

This paper is not easy to read, because the fluency and structure of written English, suggesting for proofreading.
