# Supplementary material for: Hyperbaric oxygen in children with cerebral palsy: A systematic review of effectiveness and safety
Source: PLoS One. 2022 Oct 14;17(10):e0276126. doi: 10.1371/journal.pone.0276126 (PMC9565562; doi:10.1371/journal.pone.0276126)
Supplement: Attachment — Submitted filename: RESPONSES TO REVIEWERS_V4.docx [file pone.0276126.s008.docx]

**RESPONSES TO REVIEWERS: comments on 3^rd^ version (September 2022)**

**EDITOR**

Thank you for submitting your manuscript to PLOS ONE. After careful consideration, we feel that it has merit but does not fully meet PLOS ONE’s publication criteria as it currently stands. Therefore, we invite you to submit a revised version of the manuscript that addresses the points raised during the review process.

In spite of the negative comments of one reviewer (that nevertheless deserve consideration in your next revision), I decided to indicate only minor revision. Please follloe carefully the indications of the reviewer in finalizing your submission.

---> we thank the Editor and the Reviewers for the supplementary work, which has been performed regarding this manuscript. As asked, we have provided new answers to the Reviewers where needed. Some comments by Reviewer #3 had already been rigorously answered in our previous version and I invite anyone to check for this. We have followed all the suggestions of reformulation of the Methods section.

The minor revisions asked by Reviewer #4 have been fully realized.

**REVIEWER #3**

**General comments**

As there are a number of omissions in the design of this study, the value of this topic under review is questionable. Authors still didn’t change methods as suggested. It would be difficult to read futher.

We have strictly followed the PRISMA guidelines for systematic reviews and meta-analyses and the bodies of evidence were then appraised using the GRADE evidence rating system, which ensures that the methods and results of the present review meet the stat-of-the-art expectations.

**Abstract/ Introduction-content**

No read.

We assume that there has been no detectable issue in this section.

**Methods: Identification and selection of studies**

Information is messy. Inclusion criteria should be following as Design(missing), Participants, Intervention (missing), Outcome measures (missing)

We followed the reviewer’s demand in our previous revision. The recommendations by the reviewer have been rigorously followed. The paragraph of the Methods section now reads:

“The following inclusion criteria were used for study selection:

1) Design: observational studies, before/after studies (descriptive) and controlled trials
2) Patients: children (<18 years) with CP included in the study sample;
3) Intervention: administration of HBOT or HBA;
4) Outcomes: inclusion of an effectiveness- and/or safety-related outcome for HBOT or HBA;
5) Language: articles written in English.”

The title of the paragraph has been changed into the following: *Article selection: Identification and selection of studies.*

**Methods: Assessment of characteristics of studies**

There should be more points in this part. It would be better to have this headings and follow some subheading as below:

*Quality*: information included , but a bit messy.

*Participants:* missing

*Intervention:* messing

*Outcome measures:* missing

We have already taken this comment of the reviewer into account in our previous revision, and added the suggested organization in the Methods section. We added the following subheadings:

*“Participants:* information about participants (age, type of cerebral palsy, and functional level) were detailed.

*Intervention:* data regarding the hyperbaric intervention (content: pure oxygen or air, pressure, number and duration of sessions, total volume of the intervention) and control intervention were carefully searched through and reported.

*Outcome measures:* all types of outcome measures identified in the retained articles were analysed and could subsequently be sorted as: motor function, spasticity, sleep disorders, cognitive functions, independence, for effectiveness-related outcome, and safety.”

Besides, the title of this paragraph of the Methods section has been changed and now reads: “***Quality assessment and study appraisal: Assessment of the characteristics of included studies*** in order to provide more information about the methods used.”

**Results/ Discussion/ Conclusion: context.**

As there are so many questions in the method, results, discussion and conclusion are questionable.

We are very confident that all informations about the methods that were missing in the reviewer’s opinion have been detailed in the Results section and the associated Tables

**Readability and style.**

This paper is not easy to read, because the fluency and structure of written English, suggesting for proofreading.

We asked for a careful assessment of the English in this manuscript by a native English speaker.

**REVIEWER #4**

Reviewer #4: The comments from a previous review round have overall been appropriately addressed.

The English has been improved, however multiple mistakes or style issues remain, e.g. :
- The definition of subtypes of CP, the potential interventions to prevent the extent of lesions and impairments, and the
impact of early interventions is still work in progress. SHOULD BE CORRECTED TO ...are still work in progress.

---> We thank the Reviewer for her/his careful reading. We have changed the verb as suggested.
- Considered as an complementary --> considered as A complementary

---> We thank the Reviewer for her/his careful reading. We have changed the formulation as suggested.

- with various oxygen content and pressure --> various oxygen contents and pressures OR variable oxygen content and pressure
---> We thank the Reviewer for her/his careful reading. We have changed the adjective as suggested, into “variable”.

It is not the role of the reviewers to provide copy-editing and since a re-read by an English speaker visibly was insufficient I can only advise a round of professional copy-editing before possible publication.

---> An additional reading by a native-english speaker has been performed for this revised version.

My only comment on content is around the conclusion, which in my opinion unjustifiably keeps a door open to further research on HBOT, which is not supported by this review in any way. The authors state : Therefore, we acknowledge that some questions raised by the 15-year controversy about HBOT in CP remain unsettled, and that there is a room for improvement in the methodology of experimental studies into hyperbaric interventions in children with cerebral palsy. However, encouraging results from animal studies and/or strong evidence in other neurological patients with hypoxic/anoxic brain lesions appear necessary before clinical research in children with CP can be revitalised.

I would contend that there is no additional evidence to be sought from animal studies (that have been extensively performed) or from adult research (which has also been comprehensive) and that therefore this systematic review closes the debate.

---> we agree with the reviewer on this point. In order to have the conclusion in straight line with the discussion, we altered the final part into: “However, in the absence of evidence to be sought from animal studies (that have been extensively performed) or from adult research (which has also been comprehensive), this systematic review closes the debate and claims against further clinical research into HBOT in children with cerebral palsy”
